# The CALLY Index Is Associated with Overall Survival in Patients with De Novo Metastatic Gastric Adenocarcinoma

**DOI:** 10.3390/medicina62061124

**Published:** 2026-06-09

**Authors:** Fahri Akgül, Süleyman Can, İvo Gökmen, Gizem Bakır Kahveci, İsmail Bayrakçı, Dicle Yurdatap Koç, Ece Demirdelen, Veli Çakıcı, Bülent Erdoğan

**Affiliations:** 1 Department of Medical Oncology, Iğdır Dr. Nevruz Erez State Hospital, Iğdır 76000, Türkiye; 2 Department of Medical Oncology, Faculty of Medicine, Çanakkale Onsekiz Mart University, Çanakkale 17100, Türkiye; 3 Department of Medical Oncology, Faculty of Medicine, Trakya University, Edirne 22030, Türkiye

**Keywords:** CALLY index, metastatic gastric cancer, gastric adenocarcinoma, inflammation-based biomarker, overall survival, prognosis

## Abstract

*Background and Objectives*: Systemic inflammation, nutritional impairment, and immune dysregulation are important determinants of outcomes in advanced malignancies. The C-reactive protein–albumin–lymphocyte (CALLY) index is a composite biomarker that reflects these biological domains, but its prognostic relevance in de novo metastatic gastric adenocarcinoma has not been well defined. *Materials and Methods*: This multicenter retrospective cohort study included 234 patients with de novo metastatic gastric adenocarcinoma treated between January 2015 and December 2025. Baseline CALLY was calculated before systemic treatment. A cohort-specific CALLY threshold of 1.21 was obtained using conventional ROC analysis, with all-cause mortality status at last follow-up as the binary outcome. Survival was evaluated using Kaplan–Meier analysis and Cox proportional hazards regression. To avoid guarantee-time bias, treatment exposure variables that became known only after treatment initiation, including the number of chemotherapy cycles delivered, were excluded from the baseline Cox models. Diagnosis period was included in the multivariable model to account for treatment-era heterogeneity. *Results*: Overall, 133 patients (56.8%) were classified as low-CALLY and 101 (43.2%) as high-CALLY. Median OS was significantly longer in the high-CALLY group than in the low-CALLY group (13.9 vs. 8.6 months; log-rank *p* < 0.001). Low CALLY was associated with inferior OS in univariable analysis (HR: 1.77, 95% CI: 1.31–2.38; *p* < 0.001) and remained associated with worse OS after adjustment for baseline clinicopathological factors, first-line treatment category, and diagnosis period (adjusted HR: 1.77, 95% CI: 1.24–2.53; *p* = 0.002). The PFS difference between the CALLY groups was not statistically significant (HR: 1.15, 95% CI: 0.87–1.51; *p* = 0.326). *Conclusions*: Low baseline CALLY was independently associated with shorter OS in this retrospective cohort. These findings support CALLY as a practical candidate prognostic biomarker, while external validation and time-to-event-based cut-off assessment are needed before clinical implementation.

## 1. Introduction

Gastric cancer remains a major global health problem and is among the leading causes of cancer-related mortality worldwide, with an estimated 1.1 million new cases and approximately 770,000 deaths reported annually [1]. Despite advances in systemic therapies, outcomes remain poor, particularly in advanced disease. Patients with de novo metastatic gastric cancer represent a distinct clinical subgroup characterized by aggressive tumor biology and limited survival, highlighting the need for reliable prognostic biomarkers to improve risk stratification and guide treatment strategies [2,3,4].

Cancer progression is increasingly recognized as a process influenced not only by tumor-intrinsic factors but also by host-related systemic responses, including inflammation, nutritional status, and immune function [5,6,7]. These interrelated pathways contribute to tumor progression and treatment resistance while also reflecting the overall physiological reserve of the patient. In this context, composite biomarkers integrating these domains have gained attention, as they may provide more robust prognostic information than single laboratory parameters [8,9].

The C-reactive protein–albumin–lymphocyte (CALLY) index is an emerging composite biomarker that integrates systemic inflammation, nutritional status, and immune competence using routine laboratory parameters [10]. This index has been investigated across several malignancies and has shown prognostic relevance in gastrointestinal cancers, where low values consistently correlate with poorer survival outcomes [10,11,12,13,14,15]. However, most existing evidence is derived from heterogeneous populations, particularly localized or surgically treated cohorts, and data specifically addressing de novo metastatic gastric cancer remain limited.

Accordingly, this study aimed to evaluate the prognostic significance of the baseline CALLY index in patients with de novo metastatic gastric cancer, assess its association with overall survival after adjustment for available baseline clinicopathological factors, and explore its potential role in clinical risk stratification.

## 2. Materials and Methods

### 2.1. Study Design and Patient Population

This multicenter retrospective cohort study included patients diagnosed with metastatic gastric adenocarcinoma and followed in the Medical Oncology departments of Trakya University, Çanakkale Onsekiz Mart University, and Iğdır Dr. Nevruz Erez State Hospital between 10 January 2015 and 31 December 2025. A total of 234 patients were included. Clinical, pathological, laboratory, treatment, and survival data were retrospectively obtained from electronic medical record systems and systematically recorded using a predefined standardized data collection form.

The inclusion criteria were as follows: (i) histopathologically confirmed gastric adenocarcinoma, (ii) presence of metastatic disease at diagnosis, (iii) age ≥ 18 years, (iv) availability of complete clinical and survival data, and (v) accessible laboratory parameters required for CALLY index calculation, including C-reactive protein (CRP), serum albumin, and lymphocyte count.

The exclusion criteria included: (i) incomplete clinical or laboratory data, (ii) active infection, (iii) hematological or autoimmune disease, (iv) synchronous malignancy or a history of malignancy within the previous five years, and (v) systemic conditions potentially affecting inflammatory parameters.

### 2.2. Demographic and Clinical Data

Demographic variables included age, sex, smoking history, and alcohol consumption. Clinical variables consisted of ECOG performance status and comorbid diseases, including diabetes mellitus, hypertension, and coronary artery disease. Body mass index (BMI) was calculated as kilograms per square meter (kg/m^2^) and categorized as <18.5 kg/m^2^ or ≥18.5 kg/m^2^.

Pathological evaluation included histological subtype, tumor differentiation grade, and primary tumor localization. Metastatic dissemination patterns were separately assessed according to liver, lung, lymph node, peritoneal, bone, and brain involvement. Metastatic burden was categorized as single-site or ≥2 metastatic sites.

HER2 and PD-L1 expression were evaluated using immunohistochemical methods. HER2 status was reported using a 0–3+ scoring system, whereas PD-L1 expression was classified according to staining patterns. Microsatellite instability (MSI) status was analyzed using next-generation sequencing (NGS).

### 2.3. Laboratory Measurements

All hematological and biochemical parameters were obtained from peripheral venous blood samples collected within seven days before the initiation of systemic treatment. Laboratory analyses were performed in each participating center according to standardized operating procedures and validated through internal and external quality-control programs. For reproducibility, albumin was recorded in g/dL, lymphocyte count in 10^3^/uL, and CRP in mg/L before calculation of the CALLY index.

### 2.4. Definitions and Clinical Outcomes

The C-reactive protein–albumin–lymphocyte (CALLY) index, the primary biomarker of the study, was calculated using the following formula:

CALLY = (albumin [g/dL] × lymphocyte count [10^3^/uL])/CRP [mg/L]

Treatment response was evaluated using computed tomography (CT) imaging performed routinely every three months or earlier when clinically indicated and classified according to the Response Evaluation Criteria in Solid Tumors (RECIST) version 1.1. Response categories were defined as complete response (CR), partial response (PR), stable disease (SD), and progressive disease (PD).

Overall survival (OS) was defined as the time from treatment initiation to death from any cause. Progression-free survival (PFS) was defined as the time from treatment initiation to the first radiological progression or death from any cause. Patients without an event were censored at the date of the last follow-up.

### 2.5. Statistical Analysis

All statistical analyses were performed using IBM SPSS Statistics for Windows, Version 25.0 (IBM Corp., Armonk, NY, USA). Normally distributed continuous variables were expressed as mean ± standard deviation (SD), whereas non-normally distributed variables were presented as median (minimum–maximum). Categorical variables were summarized as frequencies (*n*) and percentages (%).

The CALLY cut-off was derived using conventional ROC curve analysis with all-cause mortality status at last follow-up as the binary outcome. The selected threshold was 1.21. ROC performance was reported using the area under the curve (AUC), 95% confidence interval (CI), sensitivity, and specificity. Because conventional binary ROC analysis does not fully incorporate censoring or a fixed survival time horizon, this threshold was considered exploratory and was used primarily for group-based survival description. As a sensitivity analysis, CALLY was also evaluated as a log-transformed continuous variable in the Cox model.

Comparisons between groups were performed using the chi-square test or Fisher’s exact test for categorical variables, as appropriate.

Survival analyses were conducted using the Kaplan–Meier method, and differences between survival curves were evaluated using the log-rank test. Median survival durations and corresponding 95% confidence intervals (CIs) were calculated for both overall survival (OS) and progression-free survival (PFS).

To evaluate the prognostic impact of the CALLY index, univariable and multivariable Cox proportional hazards regression analyses were performed. Variables with *p* < 0.10 in univariable analyses and clinically relevant baseline covariates were considered for multivariable modeling. Post-baseline treatment exposure variables, including first-line and second-line chemotherapy cycle counts, were excluded from the baseline Cox model because these variables are only observed among patients who survive long enough to receive treatment and may therefore introduce immortal-time or guarantee-time bias. Hazard ratios (HRs) and 95% confidence intervals (CIs) were reported for all regression analyses.

All statistical tests were two-sided, and a *p*-value < 0.05 was considered statistically significant.

Because of the retrospective design, no formal a priori power calculation was performed. However, the cohort included 234 patients and 189 death events, providing an event count considered adequate for the primary Cox regression analyses after limiting the multivariable model to clinically relevant covariates.

## 3. Results

### 3.1. Baseline Characteristics

A total of 234 patients with metastatic gastric cancer were included in the study. The median age was 67 years (range, 27–92), with a mean age of 66.2 ± 11.0 years. Of the patients, 72.2% were male and 27.8% were female. Overall, 56.0% of the patients were older than 65 years. ECOG performance status was 0–1 in 76.9% of patients and 2–3 in 23.1%. A body mass index (BMI) ≥ 18.5 kg/m^2^ was observed in 91.0% of the cohort. Smoking history was present in 47.4% of patients, and at least one comorbidity was identified in 61.1%.

Histopathological evaluation demonstrated adenocarcinoma as the predominant histological subtype (91.5%), whereas signet ring cell carcinoma accounted for 8.5% of cases. Tumors were classified as moderately/well differentiated in 52.1% and poorly differentiated in 47.9% of patients. The majority of primary tumors were proximally located (72.2%). Molecular analysis revealed HER2 positivity in 20.6% and PD-L1 expression ≥5 in 40.6% of patients. Seven patients (3.4%) were identified as MSI-high; however, due to the limited number of cases, no further statistical analysis was performed for this subgroup.

### 3.2. Comparison of Baseline Characteristics According to CALLY Index

**Figure 1:**
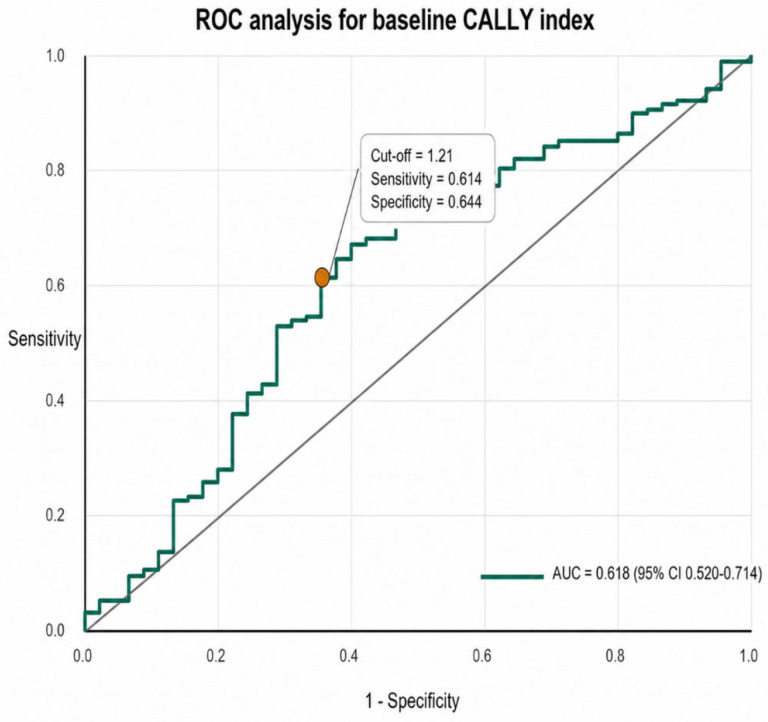
Receiver operating characteristic (ROC) curve for the baseline CALLY index using all-cause mortality at last follow-up as the outcome. The selected cut-off was 1.21, with an AUC of 0.618 (95% CI: 0.520–0.714), sensitivity of 0.614, and specificity of 0.644.

Demographic, clinical, pathological, and treatment-related characteristics according to CALLY index groups are summarized in Table 1. The optimal cut-off value for the CALLY index was determined to be 1.21. Based on this threshold, patients were categorized into low-CALLY (*n* = 133, 56.8%) and high-CALLY (*n* = 101, 43.2%) groups. ROC analysis for baseline CALLY using all-cause mortality at last follow-up as the outcome yielded an AUC of 0.618 (95% CI: 0.520–0.714). At the selected CALLY cut-off of 1.21, sensitivity was 0.614 and specificity was 0.644 (Table 2). The corresponding ROC curve is presented in Figure 1.

**Table 2:** ROC analysis summary for the baseline CALLY index cut-off.

Outcome	AUC	95% CI	Cut-Off	Sensitivity	Specificity
All-cause mortality at last follow-up	0.618	0.520–0.714	1.21	0.614	0.644

Post-baseline chemotherapy-cycle variables were excluded from the baseline Cox model to avoid immortal-time or guarantee-time bias. The multivariable model was based on complete cases for the included covariates; HER2 status was unavailable in 30 patients. Abbreviations: CALLY, C-reactive protein–albumin–lymphocyte index; CI, confidence interval; ECOG, Eastern Cooperative Oncology Group; HER2, human epidermal growth factor receptor 2; HR, hazard ratio.

No statistically significant differences were observed between the groups regarding demographic characteristics, including age and sex, or clinical parameters such as ECOG performance status, BMI, smoking history, and the presence of comorbidities (all *p* > 0.05) (Table 1).

Similarly, histopathological characteristics, including histological subtype, tumor differentiation, and tumor localization, as well as molecular parameters such as HER2 status and PD-L1 expression, were comparable between the groups (all *p* > 0.05) (Table 1). Since the number of MSI-high patients was very limited (*n* = 7, 3.4%), no subgroup comparison was performed for this parameter.

Metastatic dissemination patterns were also similar between the groups. First-line systemic treatment categories were not statistically different between the low- and high-CALLY groups (*p* = 0.689).

### 3.3. CALLY Index and Survival Analyses

**Figure 2:**
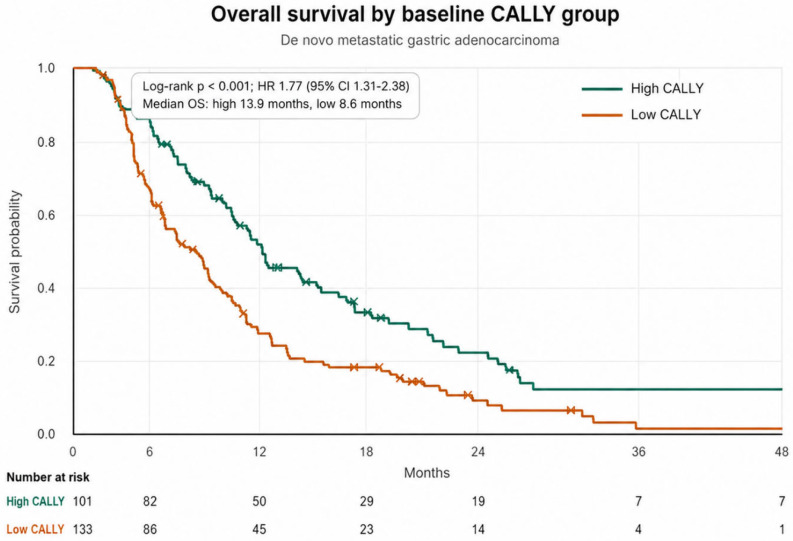
Kaplan–Meier overall survival curves according to baseline CALLY index groups in patients with de novo metastatic gastric cancer. The plot includes censored observations and the number at risk over time. Patients with low-CALLY scores demonstrated significantly poorer overall survival compared with those with high CALLY scores (log-rank *p* < 0.001; HR for low vs. high CALLY: 1.77, 95% CI: 1.31–2.38).

The median follow-up duration was 9.9 months. Kaplan–Meier survival analysis demonstrated a significant difference in overall survival (OS) between the CALLY groups (log-rank χ^2^ = 15.17, *p* < 0.001) (Figure 2). Kaplan–Meier curves showed consistently higher survival probabilities in the high-CALLY group throughout the follow-up period.

Median OS was 13.9 months (95% CI: 12.1–15.6) in the high-CALLY group, compared with 8.6 months (95% CI: 6.2–11.1) in the low-CALLY group. Mean survival durations were 21.8 and 11.9 months, respectively.

In Cox regression analyses, low CALLY was significantly associated with inferior OS in univariable analysis (HR: 1.77, 95% CI: 1.31–2.38; *p* < 0.001). In the revised multivariable baseline model, after exclusion of post-baseline chemotherapy-cycle variables and adjustment for ECOG performance status, smoking status, tumor differentiation, HER2 status, liver metastasis, peritoneal metastasis, radiotherapy, first-line treatment category, and diagnosis period, low CALLY remained independently associated with worse OS (adjusted HR: 1.77, 95% CI: 1.24–2.53; *p* = 0.002) (Table 3 and Table 4).

Regarding progression-free survival (PFS), patients in the low-CALLY group tended to have shorter PFS durations; however, this difference did not reach statistical significance (HR: 1.15, 95% CI: 0.87–1.51, *p* = 0.326).

Overall, these findings support an independent association between low baseline CALLY and poorer OS within the limits of the available retrospective dataset, whereas its association with PFS appears to be limited.

## 4. Discussion

To the best of our knowledge, this is the first study to evaluate the prognostic significance of the C-reactive protein–albumin–lymphocyte (CALLY) index in patients with de novo metastatic gastric adenocarcinoma. Low CALLY was associated with substantially shorter OS, with a median OS of 8.6 months in the low-CALLY group compared with 13.9 months in the high-CALLY group (*p* < 0.001). After revision of the baseline Cox model to exclude post-baseline chemotherapy-cycle variables and include diagnosis period, low CALLY remained independently associated with inferior OS (adjusted HR: 1.77; 95% CI: 1.24–2.53; *p* = 0.002). This finding supports an independent prognostic association, although causal interpretation is limited by the retrospective design and residual confounding.

The biological basis underlying the prognostic value of the CALLY index likely reflects the complex interaction among systemic inflammation, immune response, and nutritional status [16,17,18]. CRP is a marker of systemic inflammation associated with tumor-promoting processes, including proliferation, angiogenesis, and metastatic spread [19]. Albumin reflects both nutritional reserve and systemic inflammatory burden, while hypoalbuminemia has been associated with poor performance status and reduced treatment tolerance [20]. Lymphocyte count represents host immune competence and may indicate impaired antitumor immune surveillance when reduced [21]. Collectively, these parameters provide an integrated reflection of host physiological reserve in advanced cancer [22]. Importantly, combining these parameters within a single composite index may improve prognostic stratification by more effectively reflecting the interaction among systemic inflammation, nutritional status, and immune suppression.

The CALLY index was first introduced by Iida et al. in patients with hepatocellular carcinoma, where it demonstrated superior prognostic performance compared with conventional inflammatory and nutritional indices [23]. Since then, its prognostic significance has been investigated across several gastrointestinal malignancies. A recent meta-analysis involving gastrointestinal cancers demonstrated that a low-CALLY index was consistently associated with poorer survival outcomes across multiple oncological endpoints [24]. These findings support the prognostic utility of the CALLY index across different gastrointestinal tumor types.

Most previous studies evaluating the CALLY index in gastric cancer primarily included patients with localized disease undergoing curative-intent treatment [25,26,27,28,29]. Hashimoto et al. demonstrated that low-CALLY scores were associated with significantly worse long-term survival outcomes in patients undergoing curative gastrectomy [25]. Similarly, Aoyama et al. reported poorer survival outcomes in gastric cancer patients with low-CALLY indices who received perioperative treatment [26]. In contrast, our study specifically focused on patients with de novo metastatic gastric cancer, representing a more clinically homogeneous advanced-stage population.

Notably, Zhang et al. evaluated patients with stage I–IV gastric cancer together and reported that the prognostic impact of the CALLY index was significant mainly in stage III disease, whereas no significant association was observed in stage IV patients [12]. However, it was not clearly specified whether the stage IV cohort included patients with de novo metastatic disease or patients who developed metastatic recurrence following prior curative treatment. Therefore, direct comparison with our exclusively de novo metastatic cohort appears limited.

Another important finding of our study was the absence of a significant association between the CALLY index and progression-free survival (PFS), despite its clear relationship with OS. Similar findings have also been reported in other malignancies, including non-small-cell lung cancer [30]. This discrepancy may suggest that the CALLY index reflects the patient’s overall physiological and inflammatory status rather than the direct biological kinetics of tumor progression. While OS is influenced by host-related factors such as systemic inflammation, treatment tolerance, comorbidities, and physiological reserve [31], PFS is more closely associated with tumor response dynamics and treatment efficacy [32]. Additionally, variations in imaging frequency and treatment strategies in metastatic gastric cancer may further weaken the association between inflammation-based biomarkers and PFS.

The CALLY cut-off value of 1.21 should be interpreted with caution. It was obtained using conventional ROC analysis based on all-cause mortality status at last follow-up rather than a time-dependent ROC method at a prespecified survival horizon. This approach is simple and clinically interpretable but does not fully account for censoring in time-to-event data. Therefore, the threshold should be regarded as exploratory and cohort-specific. Reassuringly, CALLY also remained associated with OS when modeled as a log-transformed continuous variable in a sensitivity analysis (adjusted HR per one-unit increase: 0.81; 95% CI: 0.74–0.90; *p* < 0.001). Future studies should validate CALLY thresholds using time-dependent ROC methods and independent cohorts.

The potential clinical applicability of the CALLY index is also noteworthy. Since it can be easily calculated using routinely available laboratory parameters and is both inexpensive and accessible, it may help identify higher-risk patients in daily clinical practice. In particular, patients with low-CALLY scores may benefit from closer monitoring, nutritional support, and individualized treatment planning. In addition, the CALLY index may represent a promising prognostic tool for future prospective risk stratification studies.

Several limitations should be acknowledged. First, the retrospective design is inherently vulnerable to selection bias, missing data, and unmeasured confounding. Although the multicenter structure improves generalizability, center-specific practice patterns may have influenced treatment selection and outcomes. Second, the cohort spans a long treatment interval during which HER2-directed therapy, immunotherapy, molecular testing, and supportive care evolved. We adjusted for diagnosis period in the revised multivariable model, but residual treatment-era confounding may remain. Third, post-baseline treatment exposure variables, such as the number of chemotherapy cycles delivered, are susceptible to immortal-time or guarantee-time bias when entered as fixed baseline covariates; these variables were therefore removed from the revised baseline Cox model. A time-dependent modeling framework would be required to evaluate their prognostic impact appropriately. Fourth, only baseline CALLY values were available; longitudinal changes in CALLY during treatment, which may better reflect evolving inflammatory and nutritional status, could not be analyzed. Fifth, CRP, albumin, and lymphocyte measurements were obtained from three participating centers. Although laboratory values were harmonized using standardized units, inter-laboratory variation, particularly in CRP assays, may have affected CALLY reproducibility. Sixth, CALLY was not directly compared with established inflammation- or nutrition-based indices such as NLR, GPS, or PNI in the present analysis. Finally, molecular stratification was incomplete, center effects could not be fully modeled, metastatic burden was summarized using available site-based variables rather than detailed disease-volume metrics, and the ROC-derived CALLY cut-off was exploratory and cohort-specific. Prospective external validation using time-dependent cut-off methods is needed.

## 5. Conclusions

In conclusion, low baseline CALLY was associated with poorer OS in patients with de novo metastatic gastric adenocarcinoma after adjustment for available baseline clinicopathological variables, first-line treatment category, and diagnosis period. As an inexpensive and routinely available composite marker of inflammation, immune status, and nutritional reserve, CALLY may help refine risk stratification in metastatic gastric cancer. However, these results should be interpreted as an adjusted association within a retrospective dataset and require prospective validation before routine clinical use.

## Figures and Tables

**Table 1:** Baseline demographic, clinical, pathological, treatment-era, and treatment characteristics according to CALLY index groups.

Variable	Low CALLY (*n* = 133)	High CALLY (*n* = 101)	*p*-Value
Age, mean ± SD (years)	65.6 ± 11.1	67.1 ± 10.9	0.28
Male sex, *n* (%)	100 (75.2)	69 (68.3)	0.24
Smoking history, *n* (%)	58 (43.6)	53 (52.5)	0.18
ECOG 2–3, *n* (%)	27 (20.3)	27 (26.7)	0.25
BMI < 18.5 kg/m^2^, *n* (%)	11 (8.3)	10 (9.9)	0.67
Presence of comorbidity, *n* (%)	79 (59.4)	64 (63.4)	0.59
Signet ring cell carcinoma, *n* (%)	9 (6.8)	11 (10.9)	0.28
Poor differentiation, *n* (%)	65 (48.9)	47 (46.5)	0.71
HER2 positivity, *n* (%)	29/125 (23.2)	13/79 (16.5)	0.25
PD-L1 ≥ 5, *n* (%)	28/65 (43.1)	13/36 (36.1)	0.48
Liver metastasis, *n* (%)	77 (57.9)	47 (46.5)	0.08
Lung metastasis, *n* (%)	33 (24.8)	15 (14.9)	0.06
Bone metastasis, *n* (%)	19 (14.3)	24 (23.8)	0.06
≥2 metastatic sites, *n* (%)	84 (63.2)	53 (52.5)	0.09
First-line systemic treatment, *n* (%)			0.689
Fluoropyrimidine-based regimen	107 (80.5)	82 (81.2)	
HER2-based regimen	24 (18.0)	16 (15.8)	
Immunotherapy-based regimen	2 (1.5)	3 (3.0)	

HER2 and PD-L1 status were available only for a subset of patients; therefore, denominators differed across variables. Abbreviations: BMI, body mass index; CALLY, C-reactive protein–albumin–lymphocyte index; CI, confidence interval; ECOG, Eastern Cooperative Oncology Group; HER2, human epidermal growth factor receptor 2; HR, hazard ratio; MSI, microsatellite instability; PD-L1, programmed death-ligand 1.

**Table 3:** Univariate Cox regression analysis for overall survival (*n* = 234; death events = 189).

Variable	Category (Reference)	HR	95% CI	*p*-Value
Age	>65 (≤65)	1.09	0.82–1.46	0.548
Sex	Male (Female)	0.85	0.62–1.16	0.301
ECOG performance status	2–3 (0–1)	1.41	1.01–1.97	0.044
BMI	<18.5 (≥18.5)	1.21	0.72–2.02	0.479
Smoking status	Smoker (Non-smoker)	0.75	0.56–1.01	0.054
Comorbidity	Present (Absent)	0.90	0.67–1.20	0.460
Histology	Signet ring cell carcinoma (Adenocarcinoma)	1.21	0.68–2.13	0.515
Differentiation	Poor (Moderate/Well)	1.43	1.07–1.90	0.016
Tumor localization	Distal (Proximal)	1.05	0.76–1.43	0.779
HER2 status	Positive (Negative)	0.61	0.41–0.89	0.011
PD-L1 status	Positive (Negative)	1.09	0.66–1.78	0.747
Liver metastasis	Present (Absent)	1.38	1.03–1.84	0.030
Lung metastasis	Present (Absent)	1.01	0.73–1.42	0.934
Lymph node metastasis	Present (Absent)	1.01	0.76–1.35	0.944
Peritoneal metastasis	Present (Absent)	1.35	1.02–1.80	0.039
Number of metastatic sites	≥2 (1)	1.28	0.95–1.72	0.107
Surgery	Present (Absent)	0.83	0.58–1.19	0.303
Radiotherapy	Present (Absent)	0.69	0.45–1.05	0.086
First-line treatment	HER2-based regimen (Fluoropyrimidine-based)	0.64	0.43–0.97	0.033
	Immunotherapy-based regimen (Fluoropyrimidine-based)	1.98	0.73–5.39	0.181

Abbreviations: BMI, body mass index; CALLY, C-reactive protein–albumin–lymphocyte index; CI, confidence interval; ECOG, Eastern Cooperative Oncology Group; HER2, human epidermal growth factor receptor 2; HR, hazard ratio; PD-L1, programmed death-ligand 1.

**Table 4:** Revised multivariable Cox regression analysis for overall survival after excluding post-baseline chemotherapy-cycle variables and adjusting for diagnosis period (complete-case *n* = 204; death events = 164).

Variable	Category (Reference)	HR	95% CI	*p*-Value
ECOG performance status	2–3 (0–1)	1.42	0.97–2.07	0.070
Smoking status	Smoker (Non-smoker)	1.02	0.73–1.42	0.904
Differentiation	Poor (Moderate/Well)	1.52	1.08–2.14	0.017
HER2 status	Positive (Negative)	0.65	0.38–1.12	0.122
Liver metastasis	Present (Absent)	1.78	1.23–2.57	0.002
Peritoneal metastasis	Present (Absent)	1.67	1.17–2.40	0.005
Radiotherapy	Present (Absent)	0.84	0.51–1.40	0.508
First-line treatment	HER2-based regimen (Fluoropyrimidine-based)	0.96	0.54–1.73	0.897
	Immunotherapy-based regimen (Fluoropyrimidine-based)	1.11	0.34–3.63	0.867
CALLY score	Low (High)	1.77	1.24–2.53	0.002

## Data Availability

The data presented in this study are available from the corresponding author upon reasonable request.
